# Engineering of Challenging G Protein-Coupled Receptors for Structure Determination and Biophysical Studies

**DOI:** 10.3390/molecules26051465

**Published:** 2021-03-08

**Authors:** Yann Waltenspühl, Janosch Ehrenmann, Christoph Klenk, Andreas Plückthun

**Affiliations:** Department of Biochemistry, University of Zurich, Winterthurerstrasse 190, CH-8057 Zürich, Switzerland; y.waltenspuehl@bioc.uzh.ch (Y.W.); j.ehrenmann@bioc.uzh.ch (J.E.); c.klenk@bioc.uzh.ch (C.K.)

**Keywords:** G protein-coupled receptors, directed evolution, protein engineering, NK_1_R, NTS_1_R, PTH_1_R

## Abstract

Membrane proteins such as G protein-coupled receptors (GPCRs) exert fundamental biological functions and are involved in a multitude of physiological responses, making these receptors ideal drug targets. Drug discovery programs targeting GPCRs have been greatly facilitated by the emergence of high-resolution structures and the resulting opportunities to identify new chemical entities through structure-based drug design. To enable the determination of high-resolution structures of GPCRs, most receptors have to be engineered to overcome intrinsic hurdles such as their poor stability and low expression levels. In recent years, multiple engineering approaches have been developed to specifically address the technical difficulties of working with GPCRs, which are now beginning to make more challenging receptors accessible to detailed studies. Importantly, successfully engineered GPCRs are not only valuable in X-ray crystallography, but further enable biophysical studies with nuclear magnetic resonance spectroscopy, surface plasmon resonance, native mass spectrometry, and fluorescence anisotropy measurements, all of which are important for the detailed mechanistic understanding, which is the prerequisite for successful drug design. Here, we summarize engineering strategies based on directed evolution to reduce workload and enable biophysical experiments of particularly challenging GPCRs.

## 1. The Need for G Protein-Coupled Receptors (GPCRs) with Favorable Biophysical Properties

G protein-coupled receptors (GPCRs) comprise the largest family of membrane proteins and are involved in a multitude of physiological responses [1,2]. Their biological importance, combined with the ability of GPCRs to recognize a wide variety of ligands, make them highly relevant drug targets. Overall, the clinical importance of these receptors is reflected by an estimated 35% of all marketed drugs acting through a GPCR [3]. Early drug discovery programs targeting GPCRs relied solely on rather inefficient and time-consuming whole-cell screening methods, which failed to deliver hits for many important receptors. The emergence of high-resolution structures of GPCRs has finally enabled rational drug discovery programs such as structure-based drug design (SBDD) [4,5]. To date, 93 unique GPCR structures were deposited in the PDB, which were solved either by X-ray crystallography or cryo-electron microscopy (cryo-EM). Conversely, a great number of GPCRs has not yet yielded detailed mechanistic and structural studies, and some physiologically very important receptors are characterized by particularly poor expression and stability properties, making such efforts extremely challenging, if not impossible. This is the motivation behind developing engineering approaches to solve these problems.

Despite the overall advantages of structure determination based on cryo-EM and the consequent increase in structures solved with this method, structure determination by X-ray crystallography is still the best-suited method for high-throughput drug discovery programs. For cryo-EM, typically, images for a vast number of particles are recorded, from which only a small subset is used to calculate 2D class averages and 3D-reconstitutions [6,7], and the resolution given only refers to the best resolved part of the molecule, which is not necessarily the site of interest.

Meanwhile, advances in X-ray crystallography such as fast detectors, automated sample mounting and crystal soaking allow for the determination of multiple structures within one synchrotron shift [8,9,10]. However, the main drawback of X-ray crystallography remains the need for well-ordered crystals. In particular, the crystallization of membrane proteins such as GPCRs is often hampered by their low native expression levels and low intrinsic stability.

To improve the crystallization outcome for GPCRs, several techniques have been developed in the last decade to address these issues [11,12,13]. These advances include the truncation of flexible protein regions, the fusion of the receptor to small, well-folded protein partners, stabilization through conformation-specific antibodies from various species, and conformational stabilization through the introduction of thermostabilizing mutations [11,13,14,15]. Some receptors with intrinsically benign properties require only few of these measures, while others—often receptors of particular biological or biomedical interest—need to be more thoroughly engineered.

Two fundamental strategies for introducing beneficial mutations in the receptor sequence have been developed: (i) site-directed mutagenesis screening, and (ii) directed evolution-based selection. The relevance of both methods is underlined by several GPCR structures solved from receptors stabilized by one of these two methods [16,17,18,19,20,21,22,23,24,25,26].

This review will focus on GPCR engineering harnessing directed evolution. In more detail, we will summarize the currently available directed evolution-based approaches, how this engineering strategy facilitates the determination of crystal structures of GPCRs otherwise inaccessible to X-ray crystallography, the value of structures determined from these engineered receptors, and finally how engineered receptors enable other biophysical experiments for the study of receptor dynamics, in addition to crystallography, which is valuable for drug design.

## 2. Engineering GPCRs Harnessing Directed Evolution

GPCRs have evolved as intrinsically flexible proteins that sample a multitude of different conformations. They have to convey an extracellular signal, triggered by the binding of an agonist molecule, into the cytoplasm. This is achieved by the receptor undergoing a series of conformational rearrangements, moving from a ligand-free apo state over an agonist-bound intermediate state to an active state where both agonist and G protein are bound [27,28]. In the absence of an agonist, GPCRs predominantly populate a low-energy inactive “ground state”, which is probably better described as a series of similar conformations with very low energy barriers between them. In other words, the apo structure is inherently flexible, and may even populate the agonist-receiving state to a low degree, leading to a low basal activity of some receptors. Activation of the receptor by the bound agonist then leads to a shift of the conformational equilibrium, equivalent to a lowering of the energy of the higher-energy states. To efficiently populate these states, a conformational stabilization of the receptor through interaction with an agonist and a G protein is required. The flexible nature of GPCRs, inherent in its mode of action, has fundamental consequences for the determination of their molecular structure, particularly when using X-ray crystallography, but also when selecting 2D and 3D classes out of a large number of particles in cryo-EM.

Crystallization of a protein requires a high conformational homogeneity of the sample. For GPCRs, this requires a tight locking of the receptor in the desired conformation. Thus, most often, additional engineering of the protein is required to produce the diffraction-quality crystals necessary to obtain high-resolution structures [15]. A particularly successful approach has been established by introducing mutations into the wild-type receptor sequence, which enhance the detergent- and thermostability of a receptor:ligand complex. These have typically been identified through either extensive mutational screening [29,30,31,32] or directed evolution [33,34,35,36,37,38].

Site-directed mutagenesis screening, however, is a laborious undertaking, as most introduced mutations are either neutral or deleterious to various degrees for receptor expression. Thus, to retrieve the few beneficial amino acid changes, a multitude of mutations needs to be individually screened for their effect on functional expression and stability. In extensive site-directed mutagenesis approaches such as alanine scanning [29], every receptor amino acid is individually mutated, and the corresponding mutant is expressed and evaluated. When combining mutations, their effects can be additive, cancel out, or even be deleterious, requiring again an experimental screening of all combinatorial possibilities. For a single GPCR, therefore, typically roughly 300 variants have to be cloned, expressed, and characterized. Meanwhile, directed evolution allows the simultaneous screening of up to 10^8^ receptor variants, being only limited by the transformation efficiency of the recombinant host. This high capacity represents a huge advantage of directed evolution strategies compared to site-directed mutagenesis [11,34], especially in the initial phases of a project.

So far, four directed evolution approaches have been developed for GPCRs [33,36,37,38]. All four methods share the fundamental principles of directed evolution. Briefly, directed evolution mimics natural evolution and includes two main steps: (i) the randomization of a gene and, subsequently, (ii) the selection of a desired phenotype by an applied selection pressure. The directed evolution strategies discussed here work in this exact manner. First, a wild-type receptor sequence is randomized to create a library that is subsequently expressed, one receptor variant per cell. Cells displaying an increased functional receptor expression are detected and enriched by assessing the fluorescence brightness emitted by either a specially constructed receptor-fused fluorescent protein that reports on correct membrane insertion [36] or by probing functional receptor expression directly with a fluorescently labelled ligand, which can be an agonist or antagonist [33,37].

### 2.1. Escherichia Coli-Based Directed Evolution

**Figure 1:**
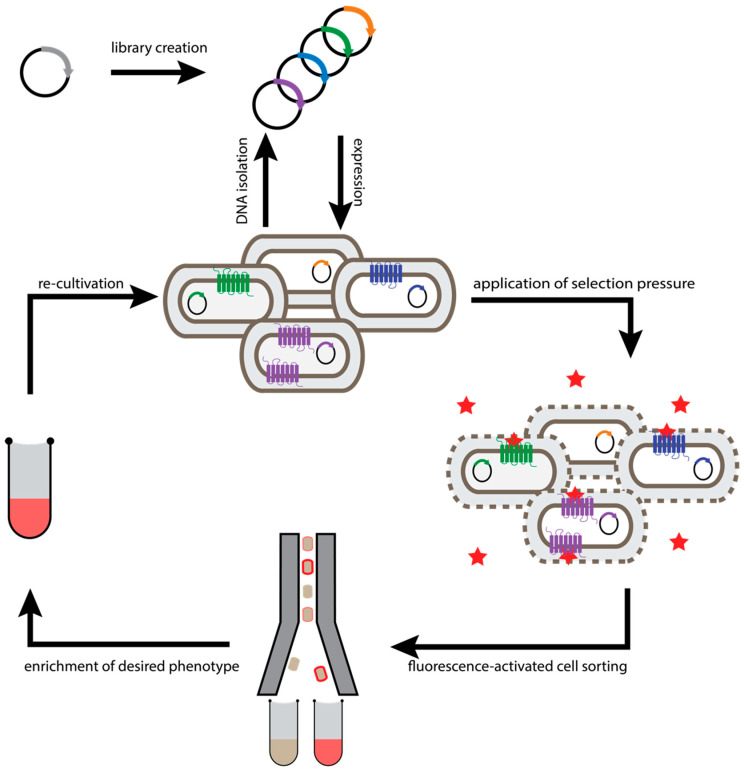
Workflow of *E. coli*-based selection methods. Evolution of GPCRs is initiated by diversification of the receptor gene. The resulting DNA library is used for transformation of *E. coli* cells, so that each cell takes up at most one plasmid molecule. Subsequently, one receptor variant is expressed in the inner membrane of a bacterial cell. To probe surface expression levels with a fluorescently labelled ligand (red star), the *E. coli* outer membrane is permeabilized (dashed oval). After incubation with fluorescently labelled ligand to bind to saturation, cell surface expression levels determine the number of bound ligands, and the cells exhibiting the highest fluorescence (i.e., cells with the highest GPCR expression) are enriched by fluorescence-activated cell sorting (FACS). Sorted cells are propagated in growth medium. Re-grown cell pools can either be subjected to additional rounds of selection or plasmids can be isolated for analysis of individual clones.

The first directed evolution method established for GPCRs was developed for the identification of GPCR variants with enhanced functional expression, stability, and binding selectivity in *Escherichia coli* (*E. coli*) [33]. While there are differences in the biosynthesis pathway of membrane proteins compared to eukaryotes (see below), the facile electroporation of *E. coli* offers a unique access to creating very large libraries. In this approach, libraries of randomized receptors are expressed in *E. coli* so that functional receptors are integrated into the inner cell membrane (Figure 1). Thereby, genotype and phenotype are linked within the cellular compartment and can later be discriminated by separation of individual cells. As an outer membrane protects the plasma membrane in *E. coli*, cells have to be selectively permeabilized to incorporate fluorescently labelled ligands into the periplasmic space to bind to the receptor. Subsequently, the best-expressing receptor variants, showing the highest fluorescence of the bound ligand, can be enriched by fluorescence-activated cell sorting (FACS) [33,34,39,40]. As functional expression levels are often linked to the biophysical properties of the receptor, many of the highly expressing receptor variants obtained by this procedure also exhibit improved thermostability [34,36]. Thus, directed evolution of GPCRs not only allows one to test millions of receptor variants in a short time, but also makes the full amino acid sequence space available to the search for advantageous mutations.

### 2.2. Generic Selection of GPCRs

All approaches to stabilize GPCRs are based on the detection of receptor integrity by ligand binding and thus require labelled ligands that specifically bind to the receptor of interest with high affinity (e.g., radioligands for stability screening of single mutants, and fluorescently labelled ligands for directed evolution and detection in FACS). However, for many receptors, these requirements are not easy to meet. Only for a small portion of all GPCRs high-affinity ligands with suitable radioactive or fluorescent labels are available, and many ligands exhibit unfavorable features that make them inappropriate for these applications. Before the structure is known, it is not always obvious where to attach a fluorescent label without compromising binding. For orphan receptors, these methods would not be applicable at all.

We therefore sought alternative ways to assess receptor integrity devoid of any specific ligands. In *E. coli*, the correct folding and integration into the plasma membrane is commonly believed to be one of the main bottlenecks for heterologous overexpression of integral membrane proteins [41,42,43]. Likewise, GPCRs, which have evolved for improved functional expression and stability, exhibit improved biophysical properties, leading to higher folding efficiency and thus to better membrane integration.

**Figure 2:**
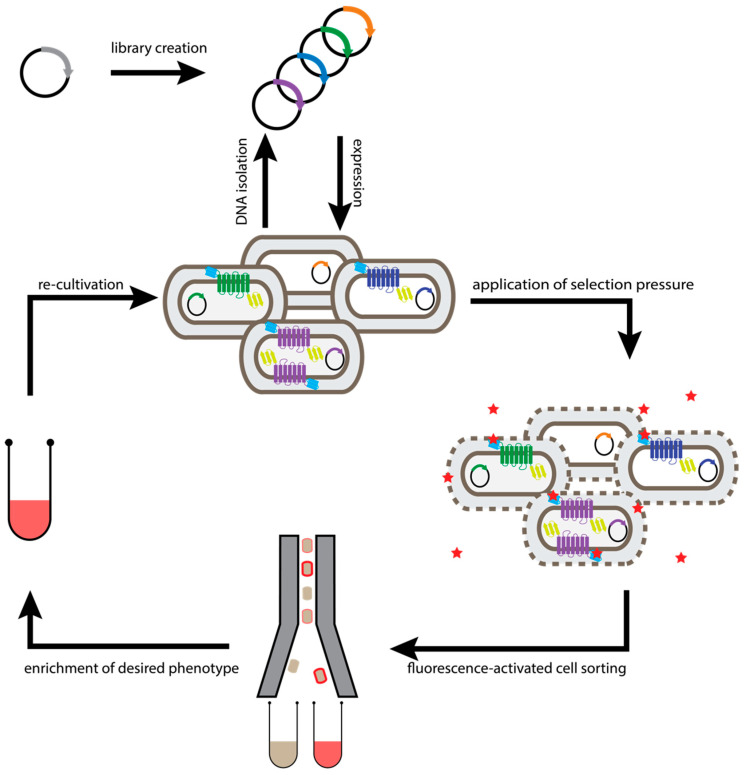
Workflow of generic selection of GPCRs. Evolution of GPCRs is initiated by diversification of the receptor gene. To enable ligand-independent assessment of surface expression, the receptor is fused N-terminally to a fluorogen-binding protein (cyan bars) and C-terminally to a fluorescent protein (yellow bars). The resulting DNA library is used for transformation of *E. coli* cells, so that each cell takes up at most one plasmid molecule. Subsequently, one receptor variant is expressed in the inner membrane of a single cell. After incubation with a fluorogen (e.g., malachite green attached to a short polyethylene glycol (PEG) molecule that makes it cell-impermeable, red star), its fluorescence greatly increases and cell surface expression levels determine the number of bound fluorophores, and the cells exhibiting the highest fluorescence (i.e., cells with the highest GPCR expression) are enriched by FACS. Sorted cells were propagated in growth medium. Re-grown cell pools can either be subjected to additional rounds of selection or plasmids can be isolated for analysis of individual clones.

Based on these findings, a selection system was developed where a small generic binding domain for a small fluorogen [44] was fused to the N-terminus, and thus at the extracellular part of the receptor. By using a fluorogen such as malachite green with a short hydrophilic polyethylene glycol (PEG) tail that was able to permeate into the periplasmic space but not to the cytoplasm, so selection of stabilized receptors in the plasma membrane was possible without the need of a specific ligand [36]. As a second selection marker, green fluorescent protein (GFP) was fused to the C-terminal end of the receptor (Figure 2). Both domains were fused to the receptor via flexible linkers. Notably, both tags were required, as the simple fusion of either the fluorogen binding domain or the GFP alone could not prevent the selection of deletion mutants, as experimentally verified [36].

### 2.3. Cellular High-Throughput Encapsulation, Solubilization and Screening (CHESS)

**Figure 3:**
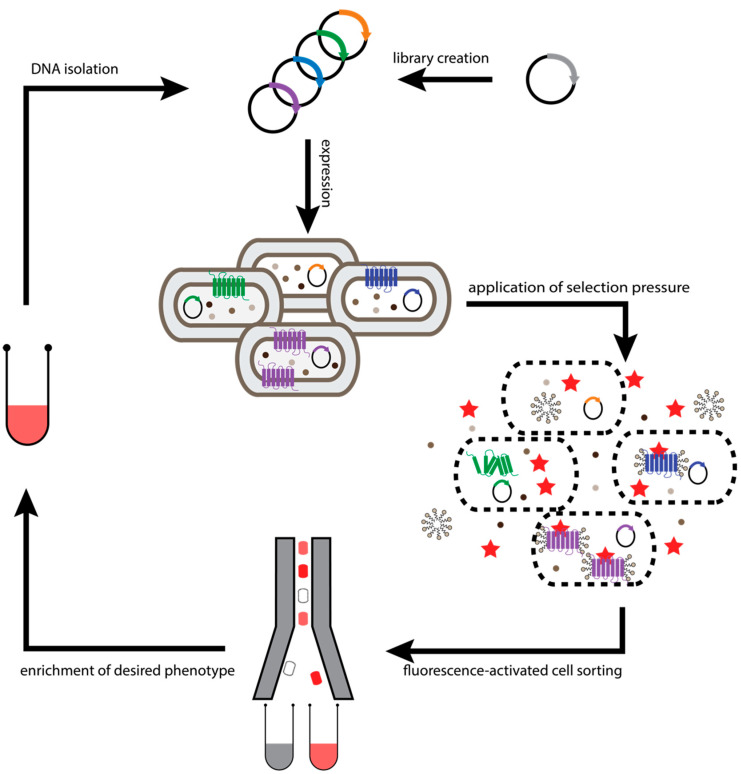
Workflow of Cellular High-Throughout Encapsulation, Solubilization and Screening (CHESS). Evolution of GPCRs is initiated by diversification of the receptor gene. The resulting DNA library is used for transformation of *E. coli* cells, so that each cell takes up at most one plasmid molecule. Subsequently, one receptor variant is expressed in the inner membrane of a bacterial cell. To probe receptor stability in detergents, the *E. coli* cells are encapsulated by several polymer layers (black dashed oval) and the cell membranes are solubilized with the detergent of choice. The polymer layers retain the shape of the original cell and convert it into a nanoscopic dialysis bag, as detergent molecules and ligands can traverse the capsule, while proteins cannot. After incubation with the fluorescently labelled ligand (red star), functional receptor levels determine the amount of fluorescent ligand retained. The capsules exhibiting the highest fluorescence (i.e., capsules containing the most stable GPCRs) are enriched by FACS. Enriched capsules of course cannot be re-grown, therefore the sequences of GPCR contained in the enriched capsules are amplified by polymerase chain reaction (PCR) and cloned into a new plasmid backbone to repeat the process or analyze individual clones.

While GPCRs that have been evolved using the bacterial display method described above typically exhibit sufficient stability in long-chain detergents, they are less robust in harsher short-chain detergents, which are typically necessary for vapor-phase crystallization and nuclear magnetic resonance (NMR) spectroscopy. To specifically increase the stability of receptors in detergents of choice, a selection system was developed where bacterial cells are coated with multiple layers of detergent-resistant alternatingly charged polymers, and then subsequently, the whole bacteria are solubilized within the capsules, with the membrane proteins becoming detergent-embedded [44] (Figure 3). Coating of the cells prevents disruption of cellular boundaries and thereby loss of the phenotype–genotype linkage. Thus, the linkage of the encoding plasmid DNA and the expressed protein is maintained. On the other hand, the polymer shell still needs to be permeable for detergents and ligands to enable solubilization and labeling of the receptor within the capsule, but the pores must not be large enough for proteins to leak out. In other words, the bacterial cell is converted to a dialysis bag of the same dimensions. The described methods have achieved this goal [44]. This has enabled the selection of several variants of the neurotensin 1 receptor (NTS_1_R) and the α_1_ adrenoceptor, specifically for retaining function when solubilized in harsh detergents. The resultant receptors were highly stable in short-chain detergents requiring no additional mutagenesis and screening after selection [38,45]. Moreover, each selection yielded receptor variants that could be crystallized readily in short-chain detergents [17,46].

### 2.4. Saccharomyces Cerevisiae-Based Receptor Evolution (SaBRE)

Despite the strong increase in functional receptor expression levels that can be achieved by directed evolution, some receptors failed in this approach, as no minimal initial expression could be obtained in *E. coli* due to high cellular toxicity of the expressed receptor construct (unpublished data). In contrast, *S. cerevisiae* is equipped with the cellular secretory quality control machinery of eukaryotes, allowing more efficient biosynthesis and translocation of complex membrane proteins. Yeast still retains the benefits of fast replication rates and high transformation efficiency, similar to *E. coli*, and most importantly, permits the creation of libraries of cells transformed with a single plasmid, which is indispensable for phenotype-genotype coupling.

**Figure 4:**
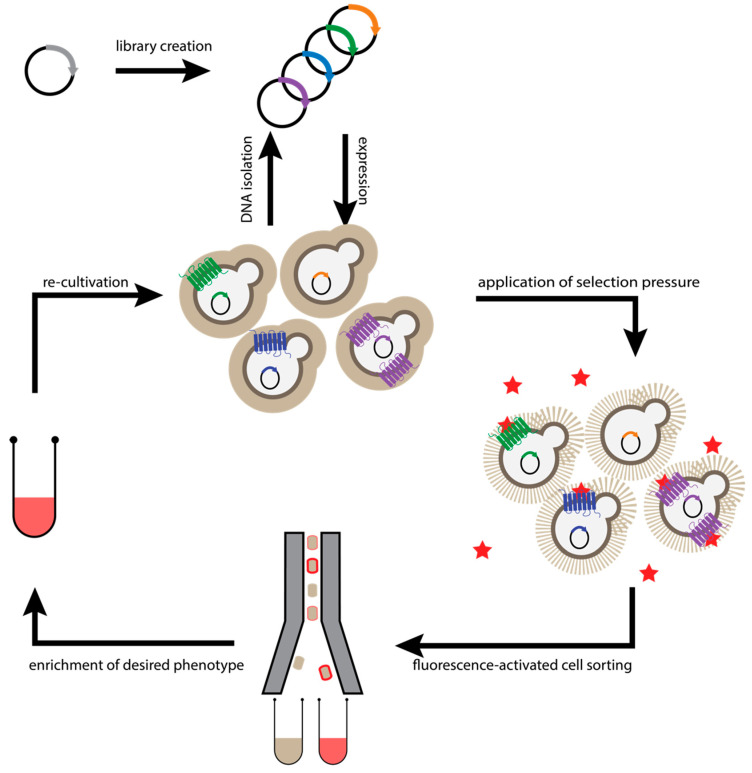
Workflow of *Saccharomyces cerevisiae*-Based Receptor Evolution (SaBRE). Evolution of GPCRs is initiated by diversification of the receptor gene. The resulting DNA library is used for transformation of *S. cerevisiae* cells, so that each cell takes up at most one plasmid molecule. Subsequently, one receptor variant is expressed in the plasma membrane of a single cell. To probe surface expression levels with a fluorescently labelled ligand (red star), the yeast cell wall was permeabilized (dashed oval). After incubation with fluorescently labelled ligand to bind to saturation, cell surface expression levels determine the number of bound ligands, and the cells exhibiting the highest fluorescence (i.e., cells with the highest GPCR expression) are enriched by FACS. Sorted cells were propagated in growth medium. Re-grown cell pools can either be subjected to additional rounds of selection or plasmids can be isolated for analysis of individual clones.

To make receptor evolution more applicable to receptor types with particularly low native expression levels, a microbial display system was devised in *S. cerevisiae* (Figure 4) [37]. Similar to the *E. coli*-based approach, high-efficiency transformation of receptor libraries was combined with cell surface display at the plasma membrane and subsequent selection of receptor variants with high functional expression using fluorescent ligands. With this approach, extremely difficult targets like the parathyroid hormone 1 receptor (PTH_1_R) or the oxytocin receptor became amenable to directed evolution, yielding well-expressing and stable receptor variants, which were the basis for determining the crystal structure of both receptors [16,18].

## 3. Insight Obtained from High-Resolution GPCR Structures

### 3.1. Advances in Understanding Class A GPCR Function

Introduction of stabilizing mutations can help to trap the receptor in a desired conformation [12,38], thus greatly facilitating structural studies of distinct receptor states. Multiple molecular structures of a receptor in different states then enables the understanding of the complex rearrangements that occur during receptor activation, as can be exemplified on the neurotensin 1 receptor (NTS_1_R).

**Figure 5:**
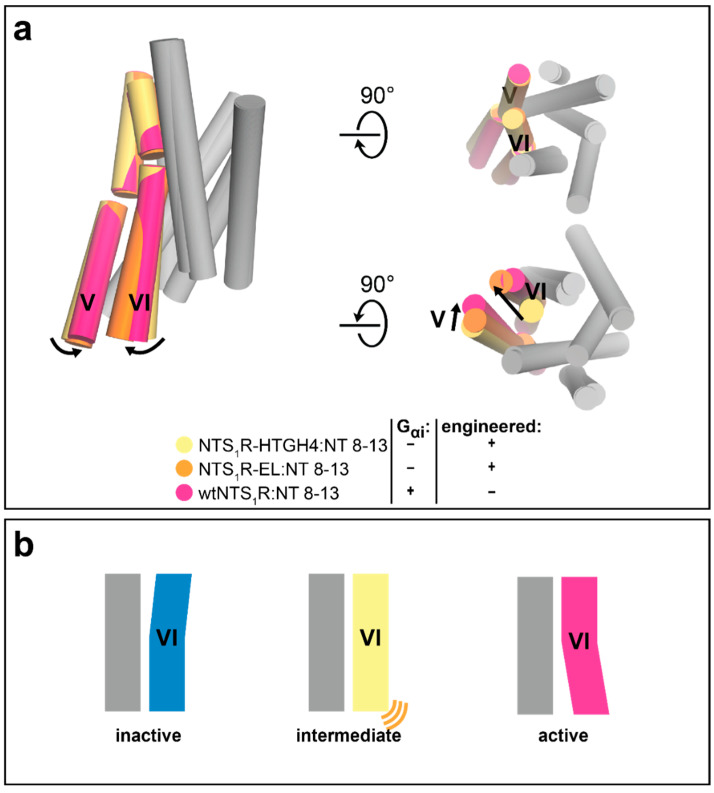
Engineered receptors are stabilized in biologically relevant states. Conformational changes during class A receptor activation. (**a**) Superposition of NTS_1_R-HTGH4 (PDB ID: 4BWB), NTS_1_R-EL (PDB ID: 5T04), and wtNTS_1_R:G_αi_ (PDB ID: 6OS9), all in complex with NT 8–13. Transmembrane helices V and VI are highlighted in color. Conformational changes are indicated by black arrows. (**b**) Schematic overview of receptor conformational states. From left: *inactive conformation* with expanded extracellular binding pocket and contracted intracellular helix bundle (blue, none of the structures above correspond to this state). *Intermediate conformation* with extracellular active-like contracted binding site, but only loosely expanded intracellular bundle (yellow helix VI, corresponding to the yellow and orange structures above). The mobility of the cytoplasmic side is indicated by orange lines. *Fully active state*, indicated by contracted binding pocket and outward movement of intracellular helix ends of helices V and VI (magenta).

The NTS_1_R has been thermostabilized in an agonist-bound state through directed evolution in bacteria [33,34,39]. Several mutants from the directed evolution process could subsequently be crystallized in complex with the C-terminal part of the endogenous agonist neurotensin (NT8-13) [17]. The structures of NTS_1_R:NT8-13 revealed the intermediate state in which the receptor had been stabilized, showing the agonist bound in the orthosteric pocket on the extracellular receptor portion, but with the intracellular receptor portion still found in an inactive conformation in the absence of a G protein (Figure 5). Interestingly, evidence for a long-lived intermediate state on the receptor activation pathway has recently been gathered through ^19^F-fluorine NMR and double electron-electron resonance (DEER) spectroscopy studies [47]. The crystal structure of the engineered NTS_1_R mutant HTGH4 in complex with an agonist, but without a G protein bound to the intracellular part of the receptor, captures exactly such a state [17].

Notably, a nearly identical intermediate conformation has also been observed in the crystal structure of a different NTS_1_R mutant (termed ELF), which had been obtained through extensive screening and subsequent combination of single thermostabilizing mutations in complex with NT8-13 [26,48] (Figure 5). The high conformational similarity between these two differently thermostabilized mutants of the NTS_1_R bound to the same ligand suggest that the stabilized conformations indeed represent actual states sampled by the wild-type receptor, rather than artificial conformations induced by the mutations, and are thus of high value for the understanding of receptor function.

Furthermore, comparison of the crystal structure of the NTS_1_R mutant HTGH4 obtained by directed evolution [17,38] with the subsequently determined structures of a constitutively active NTS_1_R mutant (EL) in complex with NT8-13 (obtained by X-ray crystallography) [49] and the fully active NTS_1_R in complex with the neurotensin-derived agonist JMV449 and a G protein (obtained by cryo-electron microscopy) [50] revealed the conformational receptor changes during late stages of receptor activation. On the extracellular receptor side, the conformation of the orthosteric pocket remained mainly unchanged, as expected when highly similar ligands are bound. On the intracellular side, the gradual outward movement of transmembrane helices (TM) V and VI can be observed from an intermediate state, in which the intracellular portion is still in an inactive conformation, to a fully active state in which the G protein is bound (Figure 5). These three receptor structures capture different conformational states and, although they represent only snapshots of local energy minima on the receptor’s energy landscape, they provide detailed information into the complex rearrangements that happen during receptor activation.

### 3.2. Insights into Receptor–Ligand Interaction

**Figure 6:**
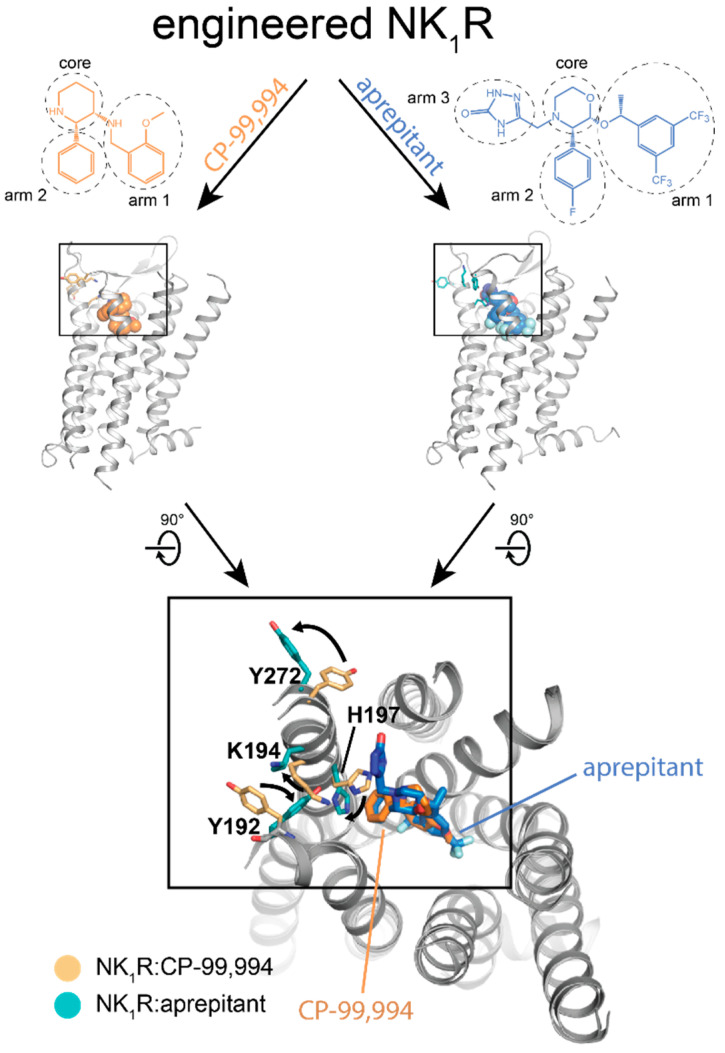
Engineered receptors allow detailed insights into GPCR ligand interactions. Conformational changes explaining insurmountable antagonism of aprepitant. Superposition of NK1R:aprepitant (PDB ID: 6HLO) and NK1R:CP-99,994 (PDB ID: 6HLL). For clarification, black arrows indicate sidechain movements.

In addition to insights into the global conformational changes of the transmembrane helix bundle during receptor activation, engineered GPCR mutants have also been instrumental in revealing changes as subtle as differences in amino acid rotamers, induced by different ligands bound to the receptor. This can be exemplified by structures of an engineered neurokinin 1 receptor (NK_1_R) mutant bound to the antagonists aprepitant and netupitant, which are clinically used for the treatment of chemotherapy-induced nausea and vomiting (CINV), and the progenitor antagonist CP-99,994 [19] (Figure 6). In contrast to the antagonist CP-99,994, aprepitant and netupitant showed insurmountable antagonism on NK_1_R, thus enabling long-lasting therapeutic effects. The crystal structures of NK_1_R in complex with these three antagonists revealed a distinct receptor conformation induced by both insurmountable antagonists, but not by their precursor CP-99,994, which did not show this effect. While the global receptor conformation remained nearly unchanged, the rotamer orientations of specific amino acid residues inside the orthosteric pocket induced by the two insurmountable antagonists aprepitant and netupitant were different from those observed in the CP-99,994-bound structure (Figure 6). These changes translate into a slight movement of ECL2 of the receptor together with the formation of a hydrophilic interaction network between the extracellular ends of TM IV and V, thus locking the receptor in a distinct conformation. Notably, the side-chain orientations of the amino acids involved in receptor–ligand interaction in this region of the receptor were identical in the aprepitant- and netupitant-bound structure, although the involved ligand moieties were very different in their chemical structure and even targeted different subpockets of the NK_1_R. Interestingly, however, all three structures of the NK_1_R have been determined from receptor:ligand complexes using the same engineered mutant. The conformational differences observed are thus induced exclusively by the bound ligand, which demonstrates that such thermostabilized receptor variants are only minimally affected by their mutations and are still capable of adopting the functionally important states.

### 3.3. Advances in Understanding Class B GPCR Function

Molecular structures have also greatly contributed to the understanding of class B GPCRs, which comprise a topology and activation mechanism distinct from that of class A receptors such as the NTS_1_R or the NK_1_R described above. Class B GPCRs share a two-domain architecture with an N-terminal extracellular domain (ECD) that is critically involved in binding the endogenous peptide ligands of these receptors. Furthermore, class B receptor activation is associated with an opening of the orthosteric binding pocket necessary to accommodate the large peptide ligands, rather than the class A receptor-typical contraction of the orthosteric pocket. However, this extracellular opening of the orthosteric pocket in class B GPCRs still translates into the canonical intracellular opening of the transmembrane domain (TMD) to allow G protein binding.

Recently, structural studies on the glucagon receptor (GCGR) in combination with kinetic measurements [51] as well as computation of the free-energy landscape associated with the activation of the receptor:agonist complex [52] have demonstrated that agonist binding alone is insufficient to promote TM VI opening at the cytoplasmic face in class B receptors. Only when in addition to an agonist also a signal-transducing G protein is bound, these receptors are able to adopt a fully active conformation. These findings can be explained by a high energy barrier that needs to be overcome in class B GPCRs to allow the simultaneous opening of the receptor TMD on the extracellular receptor portion, where the large peptide ligand is bound, and the intracellular side, where the G protein is accommodated. These conformational changes, however, require drastic rearrangements in the protein secondary structure including breaking TM VI at the conserved mid-helical kink region and unwinding its extracellular portion of TM VI [51,53,54,55,56,57,58,59,60,61]. Thus, to be able to adopt a receptor conformation that is both opened extra- and intracellularly, class B GPCRs require additional stabilization through a bound G protein.

We have recently determined a crystal structure of the class B GPCR PTH_1_R engineered for stability in an agonist-bound state independent of a bound G protein. Its crystal structure could be obtained in complex with an engineered peptide agonist (ePTH) in the absence of a G protein, and thus for first time, shed light on an intermediate state in the class B GPCR activation pathway. Previous studies of other class B receptors were all either based on inactive-state crystal structures of antagonist-bound receptor complexes or active-state cryo-EM structures of entire agonist- and G protein-bound receptor complexes.

**Figure 7:**
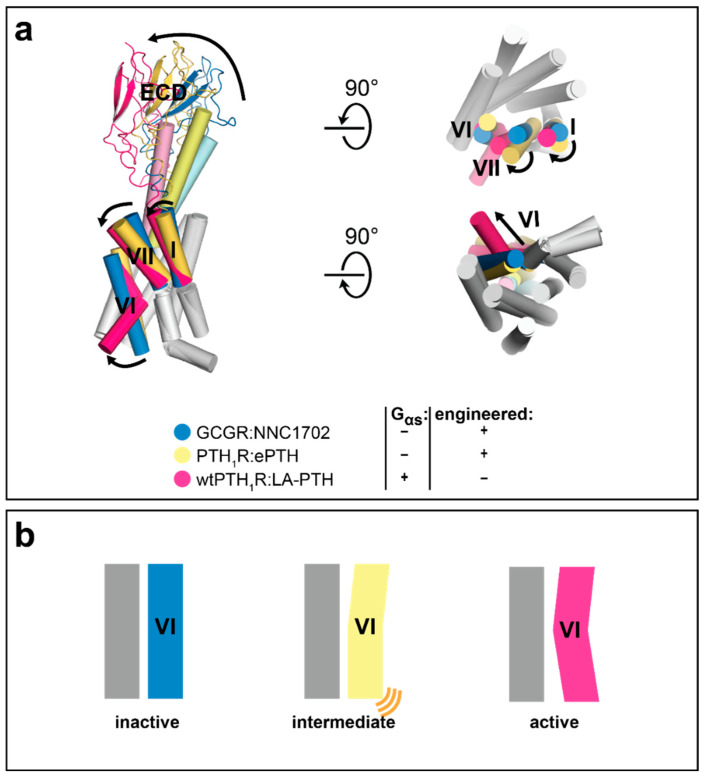
Crystal structure of engineered PTH_1_R is stabilized in an intermediate state. Conformational changes during class B receptor activation. (**a**) Superposition of GCGR (PDB ID: 5YQZ), PTH_1_R (PDB ID: 6FJ3), and wtPTH_1β_R:G_αs_ (PDB ID: 6NBF). Transmembrane helices I, VI, and VII are highlighted in color. Conformational changes are indicated by black arrows. (**b**) Schematic overview of receptor conformational states. From left: *inactive conformation* with contracted intracellular and extracellular helix portions. *Intermediate conformation* with extracellular active-like expanded binding site, but only loosely expanded intracellular helix bundle (indicated by orange lines). *Fully active state*, indicated by expanded binding pocket and outward movement of intracellular ends of helix VI.

Structural superposition of the crystal structure of PTH_1_R:ePTH with the inactive-state crystal structure of antagonist NNC1702-bound GCGR [62] and the subsequently published cryo-EM structure of fully-active G_αs_-bound PTH_1_R in complex with a different engineered agonist (LA-PTH) [60] highlights the intermediate activation state of the PTH_1_R:ePTH complex: the extracellular receptor portion is already found in an active-like conformation, while the intracellular part of the receptor still displays the hallmarks of an inactive conformation (Figure 7). The N-terminus of the agonist ePTH partly induces the clockwise rotation of the extracellular helix tips of helices TM I, TM VI, and TM VII underlying receptor activation. Due to the resulting intermediate conformation of the transmembrane helix bundle, the orientation of the ECD relative to the TMD is found midway on the activation pathway (Figure 7). As this intermediate conformation is likely the conformation to which the agonist binds before the G protein is engaged, the crystal structure of PTH_1_R:ePTH is of great relevance for the future development of new agonistic compounds.

## 4. Engineered Receptors Outside Crystallography

### 4.1. Biophysical Techniques for Structural and Functional Studies

The previously discussed high flexibility is a key feature of GPCRs, which are required to sample multiple conformations during the transition from the inactive to the active state. Ligands exert their action on a receptor by shifting the conformational equilibrium to favor a certain state, which dictates their effect as agonist, antagonist, or inverse agonist [63]. While structures obtained by crystallography can give high-resolution details on receptor–ligand interactions, they are less suited to study the dynamics of receptor activation, because they typically represent a single snapshot of the protein in a distinct state. In contrast, NMR allows quantifying receptor dynamics down to the level of side chain rearrangements and, therefore, can provide important structural information about the process of receptor activation and signal transduction.

However, recording of high-quality NMR spectra from GPCRs requires continuous measurements at elevated temperatures, which is usually not possible with native GPCRs due to their low thermostability. Using directed evolution, GPCRs can be evolved to remain functional at high temperatures for prolonged times, even after solubilization in detergents [33,34,36,39,40]. Protein stability can be increased even further by applying CHESS, which has led to ultra-stable receptor variants that remain functional in detergent micelles at more than 45 °C for weeks [38,45]. Such evolved receptor variants have been successfully used to study ligand selectivity and receptor dynamics of NTS_1_R and α1 adrenoreceptors [64,65,66,67].

For determining high-resolution protein dynamics or carrying out structure calculations by NMR, the assignment of chemical shifts is a critical step. Ideally, this is accomplished by uniform isotope labeling, which can be achieved best in *E. coli* expression systems. However, most GPCRs cannot be expressed in bacteria in their native form and thus rely on expression systems in insect or mammalian cells. This complicates protein assignment, as uniform labeling is hard to achieve in these organisms and assignment has typically been restricted to single residues obtained by selective labeling or by mutagenesis [68,69,70,71,72,73,74]. GPCRs evolved for improved functional expression in *E. coli* can reach more than 50-fold improved expression levels [34] and thus offer the opportunity to perform uniform isotope labeling strategies directly in *E. coli*. This has recently been demonstrated for an evolved variant of α_1B_ adrenoceptor [38,75]. In contrast to other stabilization approaches, evolved receptors typically do not require additional proteins fusions, which are inserted into intracellular loops to restrict protein flexibility and maintain stability. Therefore, such evolved receptor variants can even be used to study the dynamics of receptor-G protein interaction by NMR [76].

Similarly, stabilized GPCRs can be used for native mass spectrometry (nMS) to interrogate their interaction with ligands or with downstream effectors. nMS requires preserving proteins and protein complexes in their native state in solution, followed by careful transfer into the gas phase in the vacuum of the mass spectrometer. Using a stabilized variant of purinergic receptor P2Y_1_, Yen et al. assessed for the first time ligand binding by nMS with a GPCR [77]. The influence of lipids on G protein coupling selectivity was demonstrated for three different thermostabilized GPCRs using nMS [77], whereas different conformational states of a stabilized β_1_ adrenergic receptor in complex with G protein mimics were assessed by nMS, suggesting that this method is suitable for drug screening purposes [78].

### 4.2. Drug Screening

GPCRs contain ligand-binding and allosteric modulatory sites on the extracellular face of the receptor, which are accessible to pharmacological agents. Approximately half of the roughly 800 GPCR family members are being considered as potential drug targets [79,80]. From high-throughput screening of large compound libraries using cell-based assays, it is usually very difficult to find compounds with sufficient potency or selectivity, or when a rare hit has been found, it often cannot be developed further because of liabilities in the molecule [81]. Moreover, as many screening assays are based on measurements of either ligand displacement or functional responses, rather than direct observation of ligand binding, identification of novel allosteric binding sites is mostly precluded.

In recent years, several alternative biophysical methods have emerged to first directly determine the mere binding of compounds or fragments, as opposed to inducing a biological effect in a cellular assay, or displacement of a known tool compound. A direct binding measurement offers the chance of discovering new chemical matter and new allosteric pockets, but it requires access to a purified receptor of sufficient stability. Once this is in place, a protocol similar to soluble targets can be used. In this approach, the binding and dissociation kinetics and/or equilibrium affinity of ligands for GPCRs are determined, which has proven to be very useful in high-throughput drug screening campaigns.

Surface plasmon resonance (SPR) is a label-free method that measures the direct binding of a ligand with the receptor, after immobilization of the functional receptor on a chip surface. SPR enables measurements of association and dissociation rates, and as such, kinetics and affinities can be assessed simultaneously. Moreover, with SPR, molecules binding with low affinity can be detected, enabling fragment-based screening and thus offering an enhanced chemical space for screening. SPR measurements have been conducted with a few native GPCRs of the chemokine receptor family, which were isolated from cell membranes or from crude cell lysates. However, these analyses in general suffered from low receptor stability, thus only permitting a limited number of consecutive measurements. Moreover, native receptor preparations often contain high amounts of inactive receptor species and additional cellular components, reducing sensitivity, and specificity of the measurements, thus requiring extensive controls, different conditions, and orthogonal experiments [82,83,84].

In contrast, thermostabilized GPCRs are stable in a variety of detergents and can be isolated to high yields and purity, which makes them perfectly suited for in vitro measurements after surface immobilization on an SPR chip. The advantage of thermostabilized receptors for SPR screenings has initially been demonstrated on adenosine_2A_ and β_1_ adrenergic receptors, which had been stabilized by alanine scans [85,86,87]. This was followed by a study on NTS_1_R stabilized by directed evolution, which enabled fragment screening using a combination of SPR and TINS (see below) [88]. This work demonstrated for the first time the applicability of this method for peptide receptors. More recently, Ranganathan et al. [89] identified 13 new scaffolds for NTS_1_R using a combined virtual screening of fragment and lead-like libraries, encompassing more than two million compounds in total, followed by structure-based hit improvement and subsequent SPR hit validation. In a subsequent study with thermostabilized NTS_1_R, seven novel compounds were identified from a library screen by a combination of HT fluorescence polarization and SPR. Here, antagonists and agonists were identified, targeting ortho- and allosteric receptor sites [90].

Aside for structural studies, NMR-based methods can also be used to perform high-throughput compound screens. In Target Immobilized NMR Screening (TINS), binding is detected by measuring 1D-NMR spectra of compound mixtures with up to 10,000 members in the presence of a target immobilized on a solid support and comparing them to a control sample [91]. Similar to SPR and other NMR approaches, receptor stability is critical for the screening success, as measurements can take up to several days. Therefore, these methods require receptor stabilization [86]. Screening of compound libraries with TINS has initially been demonstrated with thermostabilized adenosine_2A_ and β_1_ adrenergic receptors [86,92]. Recently, NMR shift perturbation of the ligand has been used in combination with SPR for a compound screen with stabilized NTS_1_R obtained from directed evolution [88].

## 5. Conclusions

**Figure 8:**
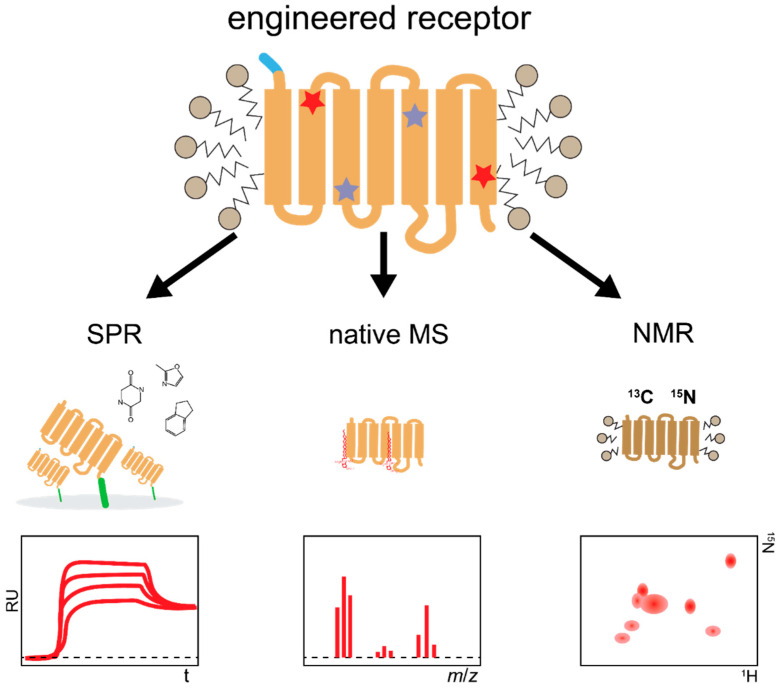
Applications of engineered receptors outside crystallography. Possible applications of receptor variants engineered for stability and enhanced functional expression. Thermostabilized receptors are well suited for methods requiring long half-life in detergent such as surface plasmon resonance (SPR) or nuclear magnetic resonance (NMR). Schematically, mutations introduced are indicated by red and purple stars. Purification tag fused to the receptors is depicted in light blue.

Due to their biological function as signal mediators, GPCRs are only scarcely present on the plasma membrane in native tissues. Their low native abundance often correlates with poor functional expression yields in recombinant hosts, drastically hampering structure determination, which requires access to pure protein. In addition, the dynamic, flexible nature of GPCRs, inherent in their mechanism of action, is the underlying cause of the often-observed native receptor instability, hampering the crystallization process. In this review, we have summarized how directed evolution-based engineering approaches offer a fast-forward platform to enable the structure determination of even the most challenging members of the GPCR family. While these techniques rely on introducing mutations into the receptor sequence, structures of engineered receptors nonetheless are highly valuable, as demonstrated by engineered receptors giving insights into the conformational states that GPCRs sample, in particular the intermediate state observed in the crystal structures of the agonist-bound NTS_1_R and PTH_1_R. Structures with different stabilizing mutations were found to be virtually identical [17] and compounds identified with the stabilized receptor were later shown to demonstrate agonist activity on the wild-type receptor [88,89]. Furthermore, structures of engineered receptors can also resolve a drug’s mechanism of action, as shown in the case of NK_1_R, whose structure was determined in complex with aprepitant and CP-99,994. Finally, engineered receptors not only provide an excellent basis for crystallization success, but are simultaneously well suited for biophysical experiments including methods such as SPR, NMR, and MS, which are challenging for unstable wild-type receptors, but invaluable to uncover receptor dynamics (Figure 8). In a broader perspective, the directed evolution approaches described herein can likely be extended to non-GPCR proteins, notably those with tight-binding ligands whose binding sites are accessible from the extracellular side. These technologies may thus also facilitate structure determination and biophysical experiments of other integral membrane protein classes.

## Data Availability

Data sharing is not applicable to this article.
